# American Medical Literature Abroad

**Published:** 1860-05

**Authors:** 


AMERICAN MEDICAL LITERATURE ABROAD.—We may be pardoned for copying the following paragraph from our little contemporary, the *Medical News and Library* for April, 1860:—

"The unusual activity of the medical press of this country within the past year, is attracting to our literature a much larger share of attention abroad than it formerly commanded. In looking over our recent files of English journals, we observe that Dr. Simpson, (whose lectures we are republishing in this periodical,) in a recent clinic on ovariotomy, alludes to the late work of Professor Gross, of this city, as the 'most complete work on systematic surgery in the English language.' The elaborate treatise on therapeutics by our fellow-townsman, Dr. Stillé, receives a short but highly complimentary notice in the London *Lancet* of March 10th; and Mr. Parrish's Practical Pharmacy is 'cordially recommended,' by the same periodical, 'as admirably suited to the requirements of the practical pharmaceutist.' The *Dublin Quarterly*, likewise, in a review of Professor Flint's valuable work on the heart, declares its 'most hearty approval of the author's ability, industry, and conscientiousness.'"

